# Non-tumor tissue derived interleukin-17B activates IL-17RB/AKT/β-catenin pathway to enhance the stemness of gastric cancer

**DOI:** 10.1038/srep25447

**Published:** 2016-05-05

**Authors:** Qingli Bie, Caixia Sun, Aihua Gong, Chunye Li, Zhaoliang Su, Dong Zheng, Xiaoyun Ji, Yumin Wu, Qi Guo, Shengjun Wang, Huaxi Xu

**Affiliations:** 1 Department of Immunology, School of medicine, Jiangsu University, Zhenjiang, Jiangsu, PR China; 2 Department of Anesthesiology, The Affiliated Hospital of Jiangsu University, Zhenjiang, Jiangsu, PR China; 3 Key Laboratory of Laboratory Medicine of Jiangsu Province, School of Medicine, Jiangsu University, Zhenjiang, Jiangsu, PR China

## Abstract

Inflammation is a critical component involved in tumor progression. Interleukin-17 (IL-17) belongs to a relatively new family of cytokines that has been associated with the progression of cancers. However, the role of IL-17B/IL-17RB (IL-17 receptor B) signaling to stemness of gastric cancer remains unknown. Here, we confirmed that the expression of IL-17RB in gastric cancer tissues was significantly increased, that overexpression was associated with poor prognosis of gastric cancer patients, and that overexpression was positively correlated with some stemness markers. Interestingly, the expression of IL-17B was upregulated in patient serum rather than gastric tumor tissues. Furthermore, exogenous rIL-17B significantly promoted the stemness of gastric cancer cells depending on IL-17RB and induced the expression of IL-17RB. Simultaneously, the expression of phosphorylated AKT, GSK-3β, and β-catenin as well as the nuclear translocation of β-catenin were significantly increased in the MGC-803 cell in a dose-dependent manner, when treated with rIL-17B. The AKT inhibitor, LY294002, and the knockdown of AKT expression reversed the rIL-17B-induced upregulation of β-catenin and some stemness markers. Together, our results indicate that the IL-17B/IL-17RB signal can promote the growth and migration of tumor cells, and upregulate cell stemness through activating the AKT/β-catenin pathway in gastric cancer, suggesting that IL-17RB may be a novel target in human gastric cancer therapy.

Gastric cancer is the fourth most common cancer and second leading cause of death from malignancy worldwide^1^. Persistent inflammation is associated with increased risk of gastric cancer^2^. Early steps in gastric carcinogenesis involve the production of proinflammatory cytokines, including IL-1β, TNFα, IFNγ, and IL-6^3^. IL-17 plays important pathogenic functions in several inflammation related cancers^4^. Interleukin-17 promotes angiogenesis by stimulating VEGF production of cancer cells in non-small-cell lung cancer^5^ and gastric cancer invasiveness by NF-κB-mediated matrix metalloproteinases 2 and 9 expression^6^. IL-17-induced epithelial-mesenchymal transition (EMT) promotes lung cancer cell migration and invasion via NF-κB-mediated upregulation of ZEB1^7^.

IL-17 consists of six family members (IL-17A to IL-17F). The IL-17R family members (includes IL-17RA to IL-17RE) are single transmembrane proteins containing fibronectin type-III domains within an extracellular portion and a unique structural motif within the cytoplasmic tails called the SEFIR [SEF (similar expression to fibroblast growth factor genes) and IL-17R] domain. Although the IL-17R family has been identified and characterized, the physiological roles of these receptors have not yet been fully determined^8^,^9^,^10^,^11^.

Accumulating evidence has suggested a strong association between overexpression of membrane receptors and proliferation, survival, and invasive ability of cancer cells, such as the amplified epidermal growth factor receptor-2 (HER2/neu) in breast cancer^12^. Overexpression of IL-17RB in murine leukemia cells has been reported to play an oncogenic role^13^. Wen-Hwa and co-workers first reported that amplified IL-17B/IL-17RB signaling promotes breast cancer tumorigenicity by activating nuclear factor-kB, to upregulate the antiapoptotic factor Bcl-2^14^. They also reported that elevated IL-17RB expression correlated with a worse prognosis and enhanced tumor malignancy in pancreatic cancer patients^15^. We previously reported that there was an increased frequency of ILC2s which expressed IL-17RB in peripheral blood mononuclear cells of gastric cancer patients^16^, and found that IL-17RB mRNA levels in gastric cancer tissues were higher than in the matched non-cancerous tissues. However, the role and mechanism of IL-17RB in gastric cancer is unknown.

The Wnt/β-catenin signaling pathway has been implicated in the maintenance of self-renewal in various types of stem cells^17^,^18^. Abnormal activation of this signaling correlates with tumorigenesis and progression by maintaining cancer stem cells (CSCs), and plays a key role in the self-renewal of gastric CSCs^19^,^20^. Recently, Xiang *et al.* demonstrated that IL-17 produced by T-helper (Th17) cells and macrophages play important roles in promoting the self-renewal of ovarian CD133 (+) cancer stem-like cells (CSLCs) by activating the nuclear factor (NF)-κB and the p38 mitogen-activated protein kinase (MAPK) signaling pathway^21^. However, the precise contribution of IL-17B/IL-17RB signaling in maintaining CSCs remains unclear.

In the following study, we confirmed gastric cancer cells cannot autocrine IL-17B, and showed that increased levels of IL-17RB were associated with poor prognosis in gastric cancer patients. IL-17B/IL-17RB signaling promoted the upregulation of stemness and EMT formation in gastric cancer cells treated with exogenous recombinant human IL-17B (rIL-17B) by the AKT/β-catenin pathway. These findings further characterize the mechanism of gastric cancer malignancy, and provide a promising therapeutic target to inhibit gastric cancer progression.

## Results

### Elevated IL-17RB expression correlates with poor prognosis of gastric cancer

**Figure 1:**
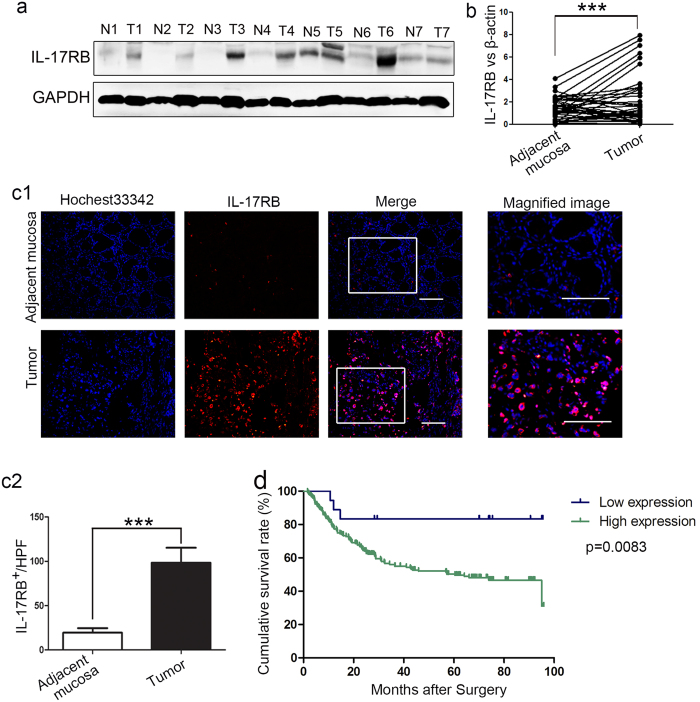
IL-17RB is overexpressed in gastric cancer (GC), and the overexpression is negatively correlated with the cumulative survival of GC patients. (**a**) Western blot analyses of IL-17RB protein expression in gastric cancer tissues and paired adjacent mucosa, the blots were cropped from the same gel. (**b**) Quantitative analyses for relative mRNA levels of IL-17RB in gastric cancer (tumor) and paired non-cancerous (adjacent mucosa) tissues. (n = 60; ***p < 0.001). The solid line designates matched samples. (**c**) Representative immunofluorescence images of IL-17RB expression showing upregulation in gastric cancer tissues. Scale bar = 50 μm. (**d**) Kaplan-Meier analysis shows a correlation between cumulative survival and IL-17RB expression levels in gastric cancer patients. Statistical significance was assessed with the log-rank test.

In order to explore the role of IL-17RB in gastric cancer, we evaluated the expression of IL-17RB protein in paired non-cancerous and gastric cancer tissues by western blot analysis. The results showed that IL-17RB was significantly increased in gastric cancer tissues (Fig. 1a). A similar expression pattern at the mRNA level was also observed using real-time quantitative PCR on 60 paired non-cancerous and gastric cancer tissues (Fig. 1b). Simultaneously, immunofluorescence staining indicated that the IL-17RB protein was predominantly expressed on the membrane of cancer cells, and that it was found in significantly higher levels in gastric cancer tissues (Fig. 1c). The correlation between IL-17RB expression and clinicopathological characteristics was further analyzed. Gastric cancer tissues with IL-17RB mRNA levels higher than paired non-cancerous tissues were defined as the high group, relative to a low group. As shown in Table 1, IL-17RB overexpression was associated with age (p = 0.037), histological grade (p = 0.002) and T categories (p = 0.05). However, no significant differences were observed between IL-17RB level and gender (p = 0.338), tumor size (p = 0.287), lymph node metastasis (N factor) (p = 0.881), or tumor node metastasis (TNM) stages (p = 0.206). To investigate whether IL-17RB expression affected the survival of gastric cancer patients, we performed immunohistochemistry (IHC) to analyze IL-17RB expression in 239 gastric cancer specimens by using a tissue microarray. The specimens were scored based on the percentage of cancer cells expressing IL-17RB, and the grading standard was shown in Fig. S1, with low expression (negative and grade I), and high expression (grade II~IV). As shown in the Kaplan-Meier survival graph (Fig. 1d), high expression of IL-17RB was correlated with poor prognosis (IL-17RB high expression versus low expression, p = 0.0083). These results suggested that IL-17RB mediated signal may affect the progression of gastric cancer.

### Non-tumor tissue derived IL-17B promotes the proliferation and migration of gastric cancer

**Figure 2:**
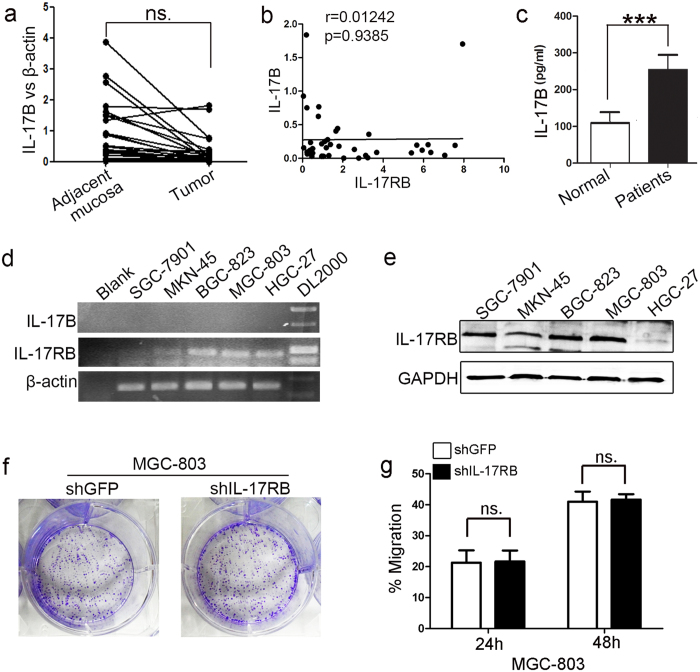
IL-17B levels are increased in peripheral blood of gastric cancer (GC) patients. (**a**) Real-time RT-PCR analyses of IL-17B mRNA levels in gastric tumors and paired adjacent mucosa (n = 60; ns, no significant). (**b**) The correlation of IL-17RB and IL-17B mRNA expression in gastric cancer tissues (r = 0.01242, p = 0.9385). (**c**) ELISA analyses the levels of IL-17B in the serum of gastric cancer patients and normal volunteers (n = 60 vs 40; ***p = 0.0003). (**d**) RT-PCR analyses of IL-17RB and IL-17B mRNA expression in SGC-7901, MKN-45, BGC-823, MGC-803, and HGC-27 human GC cells. (**e**) Western blot analyses of IL-17RB protein expression in SGC-7901, MKN-45, BGC-823, MGC-803, and HGC-27 human GC cells, the blots were cropped from the same gel. (**f**) Representative images of cell colonies in control and IL-17RB-shRNA-transfected MGC-803 cells. (**g**) The migration of IL-17RB knockdown MGC-803 cells and control cells for 24 h and 48 h was detected by using a cell scratch assay (n = 3).

Because the ligands of IL-17RB were IL-17B and IL-17E/IL-25, we quantitated the expressions of IL-17B and IL-17E mRNA in gastric cancer tissues and matched non-cancerous tissues by real-time quantitative PCR. The results showed that the expression of IL-17B in gastric cancer tissues decreased (Fig. 2a). As reported elsewhere^22^, there was no expression of IL-25 in the stomach (data not shown). In addition, we found there was no statistically significant correlation between IL-17RB and IL-17B mRNA expression in gastric cancer tissues (Fig. 2b). Wen-Hwa *et al.* confirmed that IL-17B was essential for IL-17B/IL-17RB signaling in the promotion of tumorigenicity^14^,^15^. We found that the level of IL-17B in the serum of gastric cancer patients was significantly higher than that in normal subjects, and the level of IL-25 were no significant difference, as determined by ELISA (Fig. 2c and Fig. S2c). Moreover, RT–PCR analyses showed no detectable IL-17B expression in gastric cancer cell lines (Fig. 2d), we excluded these cells autocrine IL-17B. To further confirm that the amplified IL-17RB functions in gastric cancer tissues need paracrine IL-17B, we depleted IL-17RB using its corresponding shRNA. First, we examined the expression of IL-17RB in a panel of human gastric cancer cell lines (SGC-7901, MKN-45, BGC-823, MGC-803, and HGC-27) by western blotting and RT-PCR. High IL-17RB expression was predominantly found in SGC-7901, BGC-823, and MGC-803 cells, and low expression was found in MKN-45 and HGC-27 cells (Fig. 2d,e). We then depleted IL-17RB using IL-17RB-shRNA and IL-17RB^*^-shRNA in the MGC-803 cell line that expressed high levels IL-17RB. Compared with the control cells, IL-17RB mRNA and protein levels were remarkably reduced in the IL-17RB-shRNA-transfected MGC-803 cells (Fig. S2a,b). The proliferation and migration of IL-17RB-shRNA-transfected MGC-803 cells were then analyzed using colony formation and a cell scratch assay, but no obvious differences were found compared with control cells (Fig. 2f,g). These results further confirmed that human gastric cancer cell lines do not possess an IL-17B autocrine function andIL-17B/IL-17RB signaling in gastric cancers requires paracrine IL-17B.

**Figure 3:**
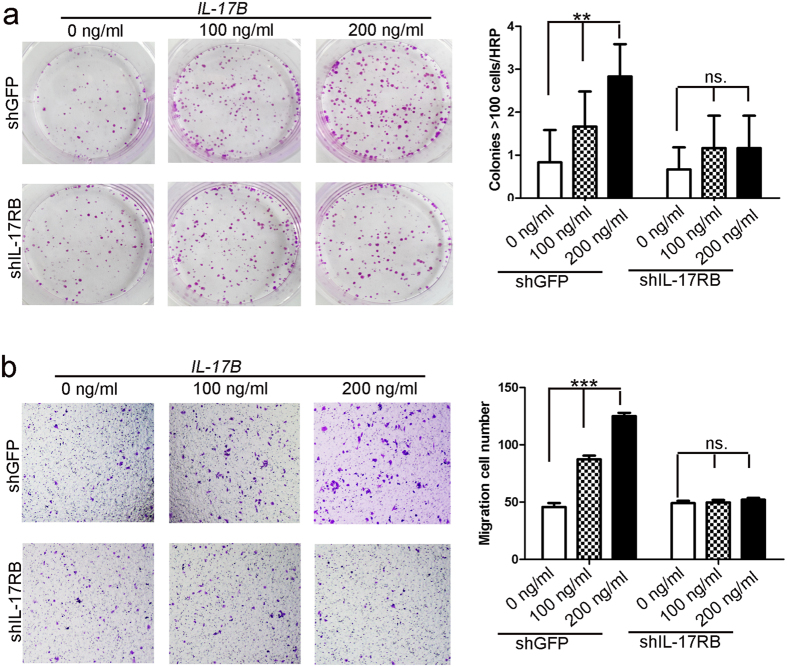
Exogenous rIL-17B promotes the proliferation and migration of MGC-803 cells by IL-17RB. (**a**) Representative images of cell colonies in control and IL-17RB-shRNA-transfected MGC-803 cells treated with 100 ng/mL or 200 ng/mL rIL-17B for 48 h. Colonies with >100 cells were quantified in three random magnifications (n = 3; **p < 0.01). (**b**) The migratory ability of control and IL-17RB knockdown MGC-803 cells treated with 100 ng/mL or 200 ng/mL rIL-17B for 48 h was evaluated by the Transwell^®^ migration assay. The number of migrated cells was quantified (n = 3; ***p < 0.001).

To further confirm the role of IL-17B/IL-17RB signaling in gastric cancer, we performed colony forming assays and Transwell^®^ assays using IL-17RB-depleted MGC-803 cells by adding exogenous recombinant human IL-17B (rIL-17B) protein. As shown in Fig. 3a,b, colony forming and migratory ability were significantly increased in a dose-dependent manner in GFP-shRNA-transfected MGC-803 control cells after exogenous rIL-17B protein was added; however, exogenous rIL-17B lost its effect when the expression of IL-17RB was deleted in MGC-803 cells. We performed colony forming assays and cell scratch assays to further confirm that the proliferation and migratory abilities were increased in a dose-dependent manner by adding exogenous rIL-17B to SGC-7901 cells (Fig. S3). Overall, these findings suggest that exogenous IL-17B combined with IL-17RB contributes to the proliferation and migration of gastric cancers.

### IL-17RB levels correlate with stemness in gastric cancer tissues

**Figure 4:**
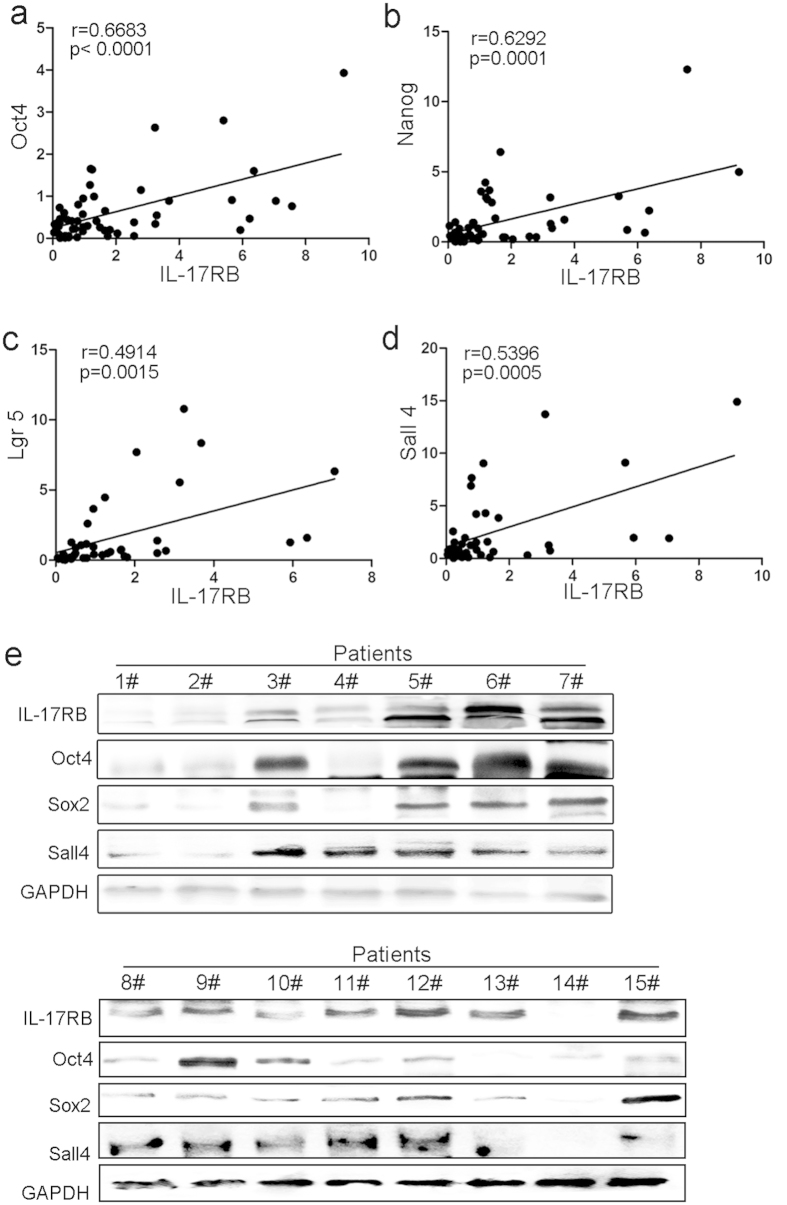
IL-17RB correlates with stemness in gastric cancer (GC) tissues. (**a**) The correlation of IL-17RB and Oct4 mRNA expression (r = 0.6683, p < 0.0001), (**b**) IL-17RB and Nanog (r = 0.6292, p = 0.0001), (**c**) IL-17RB and Lgr5 (r = 0.4914, p = 0.0015), and (**d**) IL-17RB and Sall4 (r = 0.5396, p = 0.0005). (**e**) Western blot analyses of IL-17RB, Oct4, Sox2, and Sall4 levels in gastric cancer tissues, the blots were cropped from different gels but all the gels had been run under the same experimental conditions.

During the past decade, a growing amount of evidence has suggested that CSCs play a pivotal role in various tumors. EMT in cancer cells results in the acquisition of mesenchymal traits and in the expression of stem cell markers, and is a key developmental program that is often activated during cancer invasion and metastasis^19^,^23^,^24^. And, we found rIL-17B induced MGC-803 from epithelial transition to mesenchymal morphology (Fig. S3c). To clarify whether there was a relationship between the expression of IL-17RB and stemness in gastric cancer tissues, we first detected the expression of Oct4, Nanog, Lgr5, and Sall4 mRNA using real-time quantitative PCR, and analyzed the relationship between them and IL-17RB mRNA. The results showed that there were positive correlations between IL-17RB and Oct4, Nanog, Lgr5 or Sall4 mRNA expression (Fig. 4a–d). To further confirm this result, we performed western blotting to quantitate expression of each protein in gastric cancer tissues selected randomly. Figure 4e shows that the expression of Oct4, Sox2, and Sall4 increased as the expression of IL-17RB increased. These results suggest that IL-17RB may contribute to gastric cancer cells acquiring the properties of stem cells.

### IL-17B/IL-17RB signaling induces gastric cancer stemness

**Figure 5:**
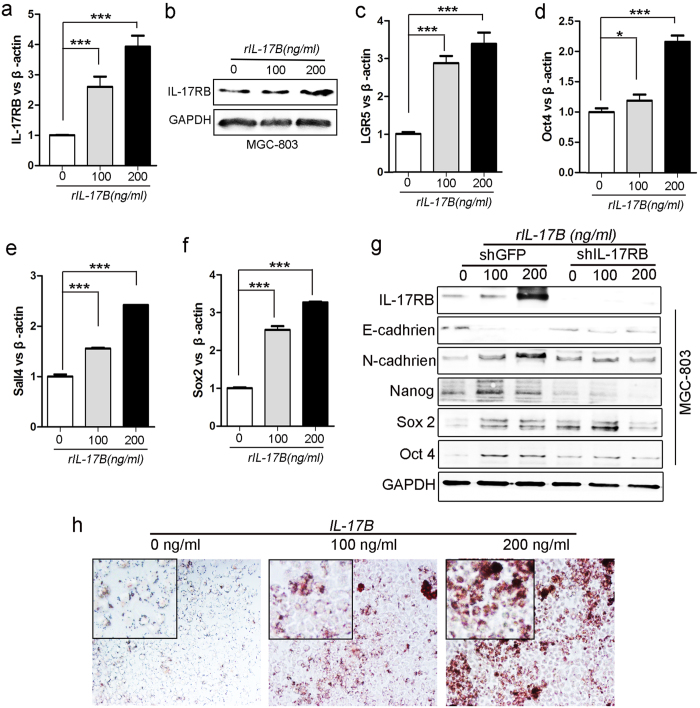
IL-17B/IL-17RB signaling induces gastric cancer (GC) stemness. Real-time RT-PCR (**a**) and Western blots (**b**) analyses of IL-17RB in MGC-803 cells treated with corresponding concentrations of rIL-17B for 48 h. Real-time RT-PCR analyses of LGR5 (**c**), Oct4 (**d**), Sall4 (**e**), and Sox2 (**f**) mRNA levels in MGC-803 cells treated with corresponding concentrations of rIL-17B for 48 h. (**g**) Western blots detecting the protein expression of IL-17RB, E-cadherin, N-cadherin, Nanog, Sox2, and Oct4 in IL-17RB knockdown cells and control MGC-803 cells treated with corresponding concentrations of rIL-17B for 48 h, the blots were cropped from different gels but all the gels had been run under the same experimental conditions. (**h**) MGC-803 cells treated with corresponding concentrations of rIL-17B for 48 h, then induced with adipogenic differentiation medium. Representative images show accumulation of lipid droplets after undergoing adipogenic differentiation.

To characterize the role of IL-17B/IL-17RB signaling on the stemness of gastric cancer, we performed real-time quantitative PCR to examine the expression of Oct4, Nanog, Lgr5, and Sall4 mRNA in MGC-803 cells treated with exogenous rIL-17B. We found that the expression of these mRNA was significantly increased in a dose-dependent manner (Fig. 5c–f). It is noteworthy that we also found the expression of IL-17RB induced by exogenous rIL-17B, increased in a dose-dependent manner in MGC-803 cells (Fig. 5a,b). Moreover, the expression of Sox2, Oct4, Nanog, and Sall4 in SGC-7901 cells treated with exogenous rIL-17B also showed a similar expression pattern (Figs S4 and S5). To further substantiate that IL-17B/IL-17RB signaling was essential in promoting upregulation of stemness-related genes in gastric cancer, we compared the protein expression by western blotting between IL-17RB-shRNA-MGC-803 cells and control cells treated with exogenous rIL-17B. MGC-803 cells transfected with IL-17RB-shRNA hardly expressed IL-17RB (Fig. 5g), which showed shRNA knockdown had no off-target effects. The expression of Nanog, Sox2, and Oct4 proteins increased in shRNA-control MGC-803 cells treated with exogenous rIL-17B, whereas exogenous rIL-17B could not increase the expression of these proteins in IL-17RB-shRNA-transfected MGC-803 cells (Fig. 5g). The expression of N-cadherin increased in a dose-dependent mannerin control MGC-803 cells and nearly had no changes in IL-17RB-shRNA-transfected MGC-803 cells in the presence of exogenous rIL-17B (Fig. 5g). However, the expression of E-cadherin decreased in control MGC-803 cells after treating with exogenous rIL-17B and nearly had no changes in IL-17RB-shRNA-transfected MGC-803 cells treated with exogenous rIL-17B (Fig. 5g). We next determined the induced differentiation potential of MGC-803 cells treated with exogenous rIL-17B, and the results showed that MGC-803 cells could be efficiently induced to differentiate into adipocytes in the appropriate conditioned medium, and adipocytes increased in a dose-dependent manner (Fig. 5h). These results suggest an important role of IL-17B/IL-17RB signaling in promoting gastric cancer stemness and EMT.

### IL-17B activates the AKT/GSK-3β/β-catenin pathway to promote stemness and EMT of MGC-803 cells

**Figure 6:**
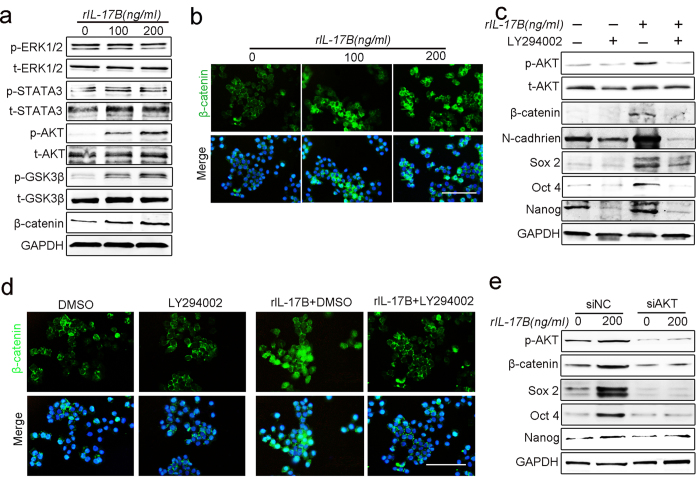
IL-17B activates the AKT/β-catenin pathway in a concentration-dependent manner. (**a**) The expressions of total and phosphorylated ERK1/2, AKT, STAT3, GSK-3β, and β-catenin were determined by western blot analyses of MGC-803 cells treated with corresponding concentrations of rIL-17B for 48 h, the blots were cropped from different gels but all the gels had been run under the same experimental conditions. (**b**) The nuclear translocation of β-catenin was detected by immunofluorescence in MGC-803 cells treated with corresponding concentrations of rIL-17B for 48 h, scale bar = 100 μm. (**c**) Western blot analysis for p-AKT and total AKT, β-catenin, N-cadherin, Sox2, Oct4, and Nanog in MGC-803 cells treated with rIL-17B (200 ng/mL) for 48 h in the presence or absence of 50 μM/mL of LY294002, the blots were cropped from different gels but all the gels had been run under the same experimental conditions. (**d**) MGC-803 cells were treated with rIL-17B (200 ng/mL) for 48 h in the presence or absence of LY294002 (50 μM/mL). The nuclear translocation of β-catenin was detected by immunofluorescence. Scale bar = 100 μm. (**e**) MGC-803cells were transfected with AKT siRNA or the matching scramble control siRNA, then western blot analysis for p-AKT, β-catenin, Sox2, Oct4, and Nanog in MGC-803 cells treated with rIL-17B (200 ng/mL) for 48 h, the blots were cropped from different gels but all the gels had been run under the same experimental conditions.

To elucidate how IL-17B/IL-17RB signaling promotes gastric cancer stemness and EMT, we analyzed the expression of proteins that could bind to the SEFIR domain within the cytoplasmic tails of IL-17RB. Western blot analysis showed that expression of phosphorylated ERK1/2 and STAT3 did not change in MGC-803 cells after exogenous rIL-17B treatment, whereas the expression of phosphorylated AKT and GSK-3β were significantly increased in a dose-dependent manner (Fig. 6a). Considering the significant role of β-catenin signaling in the self-renewal of gastric CSCs and in the induction of angiogenesis, and the effect of AKT on regulation of β-catenin signaling^20^,^25^,^26^,^27^,^28^, we also detected the expression and translocation of β-catenin in MGC-803 cells. The expression of β-catenin was consistent with phosphorylated AKT and GSK-3β (Fig. 6a). Moreover, the nuclear translocation of β-catenin in MGC-803 cells was evaluated by immunofluorescence, which increased when exogenous rIL-17B was added (Fig. 6b). To further confirm this result, we performed western blotting to measure the expression of phosphorylated AKT, total AKT, and β-catenin in SGC-7901 cell lines treated with exogenous rIL-17B. The results show that the expression of phosphorylated AKT and β-catenin also increased in a dose-dependent manner (Fig. S6).

The AKT inhibitor, LY294002, was used to characterize the IL-17B-activated AKT/β-catenin pathway in gastric cancer cells, and western blotting was used to measure the expression of β-catenin and stemness-related genes treated with or without exogenous rIL-17B. The results show that the expression of phosphorylated AKT, β-catenin, N-cadherin, Sox2, Oct4, or Nanog in MGC-803 cells treated with rIL-17B was reduced in the presence of LY294002 compared with the absence of this inhibitor (Fig. 6c). In addition, significantly reduced nuclear translocation of β-catenin was also found by immunofluorescence in MGC-803 cells treated with rIL-17B in the presence of LY294002 (Fig. 6d). Furthermore, interfering the expression of AKT by siRNA reversed the up-regulation of p-AKT, β-catenin, Sox2, Oct4 and Nanog induced by rIL-17B (Fig. 6e). Together, the results suggest that IL-17B/IL-17RB signal may induce stemness in gastric cancer cells by activating the AKT/β-catenin pathway.

## Discussion

IL-17RB (also known as IL-25R, Evi-27, or IL-17rh-l) is mainly expressed in various endocrine tissues and Th2 cells, with high expression in kidney and liver and comparable expression in the stomach^29^,^30^. IL-17RB serves as the receptor for both IL-17B and IL-17E. IL-17E (IL-25) transduces signals through a heterodimeric receptor complex composed of IL-17RA and IL-17RB, and IL-17B signals through homodimers of IL-17RB^30^,^31^. Low levels of IL-17B mRNA have been detected in several organs including adult stomach, small intestine, and pancreas, but there is no expression of IL-25 in the stomach^22^,^29^,^30^,^31^.

Previous studies have shown an oncogenic role for IL-17RB in murine leukemia cells^13^. Recently, Lee Wen-Hwa *et al.* first reported that increased IL-17RB/IL-17B signaling was associated with poor prognosis of breast cancer patients. Another study reported that overexpression of IL-17RB was correlated with postoperative metastasis and was inversely correlated with progression-free survival in pancreatic cancer patients^14^,^15^. In the present study, we found, for the first time, that expression of IL-17RB was significantly increased in gastric cancer tissues, and patients with elevated IL-17RB expression had a worse prognosis.

We found that the expression of IL-17B mRNA in gastric cancer tissues may be decreased, and had no relationship with amplified IL-17RB mRNA expression. Moreover, there was no detectable IL-17B and IL-17E (data not shown) expression in several gastric cell lines, unlike breast cancer or pancreatic cancer cell lines^14^,^15^, but the serum level of IL-17B in gastric cancer patients was significantly higher than that in normal subjects. In addition, our results indicated that the proliferation and migration of IL-17RB-shRNA-transfected MGC-803 cells showed no difference compared with control cells. When we added exogenous rIL-17B, the colony forming and migration ability were significantly increased in a dose-dependent manner in GFP-shRNA-transfected MGC-803 control cells, while exogenous rIL-17B lost its effect in IL-17RB-shRNA-transfected MGC-803 cells. Importantly, exogenous rIL-17B induced the expression of IL-17RB in MGC-803 cells and increased in a dose-dependent manner. We postulate that amplified IL-17RB in gastric cancer tissues may depend on IL-17B derived from the tumor microenvironment. Therefore, identifying potential cellular sources of IL-17B is crucial for searching for a promising therapeutic target to inhibit gastric cancer progression in future studies.

EMT has been proposed as a developmental process that plays a crucial role in cancer progression and metastases. In malignant cancer cells EMT results in the acquisition of mesenchymal traits^24^. Oct4, Nanog, and Sox2 are the core regulators of embryonic stem cell (ESC) pluripotency, and studies have reported the overexpression of Oct4 and Nanog in human gastric cancer^32^,^33^,^34^. Zhang *et al.* reported that SALL4 had oncogenic roles in gastric cancer, by modulating the EMT and cell stemness^35^, and enhanced levels of intestinal stem cell marker LGR5 was related to the malignancy of gastric cancer^36^. Our data also showed that there was a positive correlation between IL-17RB and Oct4, Nanog, Lgr5, or Sall4 mRNA expression, and that the expression of Oct4, Sox2, and Sall4 proteins increased in proportion to the increased expression of IL-17RB in gastric cancer tissues. In addition, some proteins expression levels were not positive correlation with the expression level of IL-17RB, it may be related to individual differences in tumor patients. It is necessary to consider the combined detection in tumor diagnosis when these proteins are used as indexes. The expression of Nanog, Sox2, and Oct4 proteins increased in control MGC-803 cells in the presence of exogenous rIL-17B, whereas there was no change in IL-17RB-shRNA-transfected cells. In addition, MGC-803 cells could be efficiently induced to differentiate into adipocytes that increased in a dose-dependent manner with exogenous rIL-17B. Thus, inhibiting the expression of IL-17RB may influence the expression of stemness markers through interfering with IL-17B/IL-17RB signaling.

Abnormal activation of the Wnt/β-catenin signaling pathway strongly correlates with tumorigenesis, progression, and maintenance of CSCs^19^,^20^,^25^, and the Wnt/β-catenin pathway contributes to EMT regulation in cancer cells^37^,^38^,^39^. GSK3β is a critical component of the canonical Wnt pathway, and the inactivation of GSK3β by PI3K/Akt inhibition may lead to accumulation of β-catenin in the nucleus, which is one of the hallmarks of activated β-catenin signaling in cancer^26^,^27^. Recently, Xiang and co-workers reported that IL-17 plays an important role in promoting the self-renewal of ovarian CD133 (+) cancer stem-like cells (CSLCs), by activating the NF-κB and MAPK signaling pathways^21^. Previous studies have reported that IL-17A signaling in lung epithelial cells was associated with weak AKT phosphorylation and inhibition by specific PI3K inhibitors^40^. IL-17R has been shown to transduce its signal through the activation of NF-κB, ERK, GSK3β, JAK/STAT, and MAPK pathways by a different motif^10^,^40^,^41^,^42^,^43^. We hypothesize that IL-17B/IL-17RB signaling enhances the stemness of gastric cancer by the AKT/β-catenin pathway. Our findings were confirmed by a positive correlation between IL-17RB and cell stemness. The signaling of ERK1/2 and STAT3 showed no obvious differences in MGC-803 cells treated with corresponding concentrations of exogenous rIL-17B, whereas the expression of phosphorylated AKT and GSK-3β, β-catenin, and the nuclear translocation of β-catenin were significantly increased in a dose-dependent manner. Treatment with the AKT inhibitor, LY294002, and the knockdown of AKT expression reversed IL-17B-induced upregulation of AKT/β-catenin signaling and stemness in MGC-803 cells. To our knowledge, these results are the first to show that IL-17B/IL-17RB signaling promotes gastric tumorigenesis by upregulating the stemness of gastric cancer cells through activation of the AKT/β-catenin pathway. And, we will further confirm the role of IL-17B/IL-17RB signalling on gastric tumorigenesis *in vivo* model in the future.

## Methods

### Human gastric cancer tissue specimens and patient information

The primary gastric cancer tissues and their matching adjacent noncancerous tissues (located more than 5–10 cm away from the primary site) were collected from 60 gastric cancer patients undergoing surgery at the Affiliated Hospital of Jiangsu University in Zhenjiang, Jiangsu, China. The median age of patients (42 men and 18 women) was 63.83 years (range 27–82 years). All samples were confirmed by pathological examination. Histological grade was defined according to the World Health Organization classification.

Tissue microarrays containing 239 cases with primary gastric cancer were used for detection of IL-17RB expression, which were preserved in the Gastric Cancer Tissue Bank at Department of Oncology, Changzheng Hospital (Shanghai, China). All the cases received curative resection.

### Ethics statement

The study conformed to the principles outlined in the Declaration of Helsinki and in accordance with the approved guidelines. Written informed consent was obtained from each participant prior to tissue samples collection. All samples were taken in accordance with the regulations and approval of the Affiliated Hospital of Jiangsu University and the Changzheng and Changhai Hospital Institutional Review Board.

### Cell culture and Reagents

Gastric cancer cell lines (SGC-7901, MKN-45, BGC-823, MGC-803 and HGC-27) were purchased from American Type Culture Collection (ATCC) and cultured in Dulbecco’s modification of Eagle’s medium Dulbecco (DMEM) medium containing 10% fetal bovine serum (Gibco, Grand Island, USA) at 37 °C with 5% CO_2_ atmosphere. IL-17B were purchased from R&D corporation.

### Western blotting

Tissue samples and gastric cancer cells were homogenized and lysed in RIPA buffer supplemented with proteinase inhibitors. Equal amounts of total protein were loaded and separated on 12% SDS-polyacrylamide gel. Following electrophoresis, the proteins were transferred to a PVDF (polyvinylidene difluoride) membrane (Millipore, USA), blocked in 5% (w/v) non-fat milk and incubated with the primary antibodies. Membranes were incubated with monoclonal antibody against GAPDH (CWBIO, CW0100), IL-17RB (Sigma, SAB1303013), Nanog (SAB, 21423), Oct4 (Santa Cruz, SC-5279), Sall4 (Abnova, H00057167-M03), Sox2 (Milipore, AB5603), p-ERK1/2 (Thr202/Tyr204) (Cell Signal Technology, 4370), t-ERK1/2 (Cell Signal Technology, 9102), p-AKT (Ser473) (Cell Signal Technology, 4060), t-AKT (Cell Signal Technology, 4691), p-STAT3 (Thy705) (Cell Signal Technology, 9145), t-STAT3 (Cell Signal Technology, 4904), p-GSK3β (Ser9) (Cell Signal Technology, 5558), t-GSK3β (Cell Signal Technology, 9832), β-catenin (Cell Signal Technology, 8480), N-cadherin (Cell Signal Technology, 13116), E-cadherin (Cell Signal Technology, 3195) at 4 °C overnight. The membrane was washed with Tris-buffered saline/Tween (TBS/T) for three times and incubated with the secondary antibodies (Bioworld) at 37 °C for 1 h. The signals were visualized by using Luminata crescendo western horseradish peroxidase (HRP) substrate (GE corporation, USA).

### Immunofluorescence

Formalin-fixed paraffin-embedded tissue sections were deparaffinized in xylene, rehydrated through graded ethanol and then boiled for 10 min in citrate buffer (10 mM, pH 6.0) for antigen retrieval. Slides were then blocked with 5% BSA (bovine serum albumin; Boster Bioengineering, Wuhan, China), incubated with IL-17RB antibody at 4 °C overnight and secondary antibody at 37 °C for 1 h successively, and then stained with Hochest 33342 for nuclear staining.

The cells cultured in 24-well chamber slides were washed twice with cold phosphate-buffered saline (PBS), fixed with 4% paraformaldehyde for 30 min at 4 °C, permeabilized with 0.1% Triton X-100 for 5 min, blocked with 5% BSA, incubated with IL-17RB and β-catenin antibodies at 4 °C overnight and followed by a Cy3-conjugated and FITC-conjugated anti-rabbit secondary antibody (sigma). The cells were then stained with Hochest 33342 for nuclear staining, and the images were acquired with a Nikon eclipse Ti-S microscope (Nikon, Tokyo, Japan).

### RNA extraction, RT-PCR and real-time RT-PCR

Total RNA was extracted from cells and tissues using TRIzol reagent (Invitrogen, Carlsbad, CA, USA) according to the manufacturer’s instructions, and equal amount of RNA was used for RT-PCR and real-time RT-PCR analyses. β-actin was used as an internal control. The sequences of specific primers are listed in Supplementary TableS1.

### ELISA for serum IL-17B and IL-25

Serum level of IL-17B and IL-25 was measured by ELISA kit (RB China) following the manufacturer’s protocols. All samples were measured in triplicate, and the mean concentration was calculated from the standard curve.

### siRNA transfection

Chemically synthesized AKT siRNAs and the matching scramble control siRNAs were purchased from GenePharma Company (GenePharma, Suzhou, China). Their corresponding sequences were shown in Supplementary Table S2. The siRNAs were transiently transfected into MGC-803 cells by using Lipofectamine 2000 reagent (Invitrogen) according to the manufacturer’s instructions. Cells were plated in six-well plates at a density of 1 × 10^5^ cells/well. The transfection reagent and scrambled siRNA-transfected cells were used as control.

### Lentiviral knockdown of IL-17RB in MGC-803

The specific lentiviral expression vector (sigma) containing the IL-17RB shRNA sequence was selected for specifically targeting IL-17RB silence, which was classified as Lenti- IL-17RB -shRNA, and Lenti-GFP-shRNA as negative control vector. The IL-17RB shRNA lentivirus vectors were generated by ligating the vector Tet-pLKO-puro. IL-17RB shRNA oligonucleotide sequences were shown in Supplementary Table S2. The recombinant lentivirus was produced by co-transfecting HEK293T cells with recombinant shRNA-vector, PU1562 and PU1563 plasmid using Lipofectamine 2000 (Invitrogen). The virus-containing supernatant was harvested at 48 h and 72 h post-transfection. MGC-803 cells were transduced with the prepared lentivirus (Lenti- IL-17RB-shRNA, Lenti- IL-17RB^*^ -shRNA or Lenti-GFP-shRNA). Stable cell lines were obtained after selection with 1 ug/mL of puromycin (Invitrogen) for 3 days. The expression of shRNA was induced by addition of doxycycline with 50 mg/ml for 2 days. The efficiency of IL-17RB knockdown was evaluated by using real-time quantitative PCR and western blotting.

### Colony-formation assay

Cells were harvested and seeded into six-well plate (800 cells/well) and incubated at 37 °C in a 5% CO_2_ humidified incubator for 14 days. The medium was changed at 3-days interval. At the end of the incubation period, the cultures were fixed with 4% paraformaldehyde and stained with crystal violet.

### Cell scratch assay

Cells were seeded at the density of 5 × 10^5^ cells/well in 6-well plates and incubated at 37 °C in 5% CO_2_ for 24 h to create confluent mono-layers. The mono-layers were scratched with a sterile pipette tip. To measure the mobility of cells, we obtained images in 5 random fields at 12 and 24 h after scratching. The width of original scratch was calculated by using the NIH Image program (http://rsb.info.nih.gov/nih-image/). The ratio of migration was calculated as follows: (the width of original scratch - width of actual scratch)/width of original scratch × 100.

### Transwell migration assay

Cells (1 × 10^5^/well) were plated into the top chamber and 10% FBS containing medium was placed into the bottom chamber. After incubation at 37 °C in 5% CO_2_ for 12 h, the cells remaining at the upper surface of the membrane were removed with a cotton swab. The cells that migrated through the 8-mm sized pores and adhered to the lower surface of the membrane were fixed with 4% paraformaldehyde, stained with crystal violet and photographed.

### Adipogenic differentiation *in vitro*

MGC-803 cells were seeded at the density of 5 × 10^5^ cells/well in 6-well plates and incubated at 37 °C in 5% CO2 for 24 h, then treated or not with 100ng/ml or 200ng/ml human rIL-17B for 48h. Then changed adipogenic differentiation medium (HUXUC-90031, Cyagen Biosciences, CA, USA) according to the manufacturer’s instructions. At the end of induction, adipogenic potential by oil red O staining.

### Statistical analysis

All data were shown as means ± standard deviation (SD). The statistically significant differences between groups were assessed by analysis of variance (ANOVA) or t-test using Prism software (GraphPad, San Diego, USA). P value < 0.05 was considered significant. The association between clinicopathological variables and IL-17RB expression was tested using chi-square test. Overall survival curves were plotted according to the Kaplan–Meier method, with the log-rank test applied for comparison.

## Additional Information

**How to cite this article**: Bie, Q. *et al.* Non-tumor tissue derived interleukin-17B activates IL-17RB/AKT/β-catenin pathway to enhance the stemness of gastric cancer. *Sci. Rep.* **6**, 25447; doi: 10.1038/srep25447 (2016).

## Supplementary Material

Supplementary Information

## Figures and Tables

**Table 1:** Correlation between clinicopathological factors and IL-17RB mRNA expression in gastric cancer patients.

Factor	Number (%)	IL-17RB expression		P-value
		Low group	High group	
Age(years)				
>60	39 (65%)	15	24	0.037*
≤60	21 (35%)	14	7	
Gender				
Male	42 (70%)	22	20	0.338
Female	18 (30%)	7	11	
Size(cm)				
>5	27 (45%)	11	16	0.287
≤5	33 (55%)	18	15	
Histological grade				
Well + moderately	33 (55%)	22	11	0.002**
Poorly + signet	27 (45%)	7	20	
T grade				
T1 + T2	9 (15%)	7	2	0.050*
T3 + T4	51 (85%)	22	29	
Lymph node metastasis(N factor)				
Absent(N0)	15 (25%)	7	8	0.881
Present(N1-N3)	45 (75%)	22	23	
Stage				
I/II	24 (40%)	14	10	0.206
III/IV	36 (60%)	15	21	

*P < 0.05, **P < 0.01.
