# Supplementary material for: Non-tumor tissue derived interleukin-17B activates IL-17RB/AKT/β-catenin pathway to enhance the stemness of gastric cancer
Source: Sci Rep. 2016 May 5;6:25447. doi: 10.1038/srep25447 (PMC4857095; doi:10.1038/srep25447)
Supplement: Supplementary Information [file srep25447-s1.doc]

**Non-tumor tissue derived interleukin-17B activates IL-17RB/AKT/β-catenin pathway to enhance the stemness of gastric cancer**

Qingli Bie^1*^, Caixia Sun^2*^, Aihua Gong^3*^, Chunye Li^2^, Zhaoliang Su^1,3^, Dong Zheng^1^, Xiaoyun Ji^1^, Yumin Wu^1^, Qi Guo^1,3^, Shengjun Wang^1,3#^, Huaxi Xu^1,3#^

^1^ Department of Immunology, School of medicine, Jiangsu University, Zhenjiang, Jiangsu, PR China.

^2^ Department of Anesthesiology, The Afﬁliated Hospital of Jiangsu University, Zhenjiang, Jiangsu, PR China.

^3^ Key Laboratory of Laboratory Medicine of Jiangsu Province, School of Medicine, Jiangsu University, Zhenjiang, Jiangsu, PR China.

***** These authors contributed equally to this work.

# **Corresponding author:**

Huaxi Xu, Shengjun Wang

Dept of Immunology

School of Medicine

Jiangsu University

Xuefu road 301

Zhenjiang, PR China

Phone: +86 511 88791048

Fax: +86 511 88791739

Email: [xuhx@ujs.edu.cn](mailto:xuhx@ujs.edu.cn)

**Running title:**  IL-17B/IL-17RB signaling pathway in gastric cancer

**Supplementary materials**


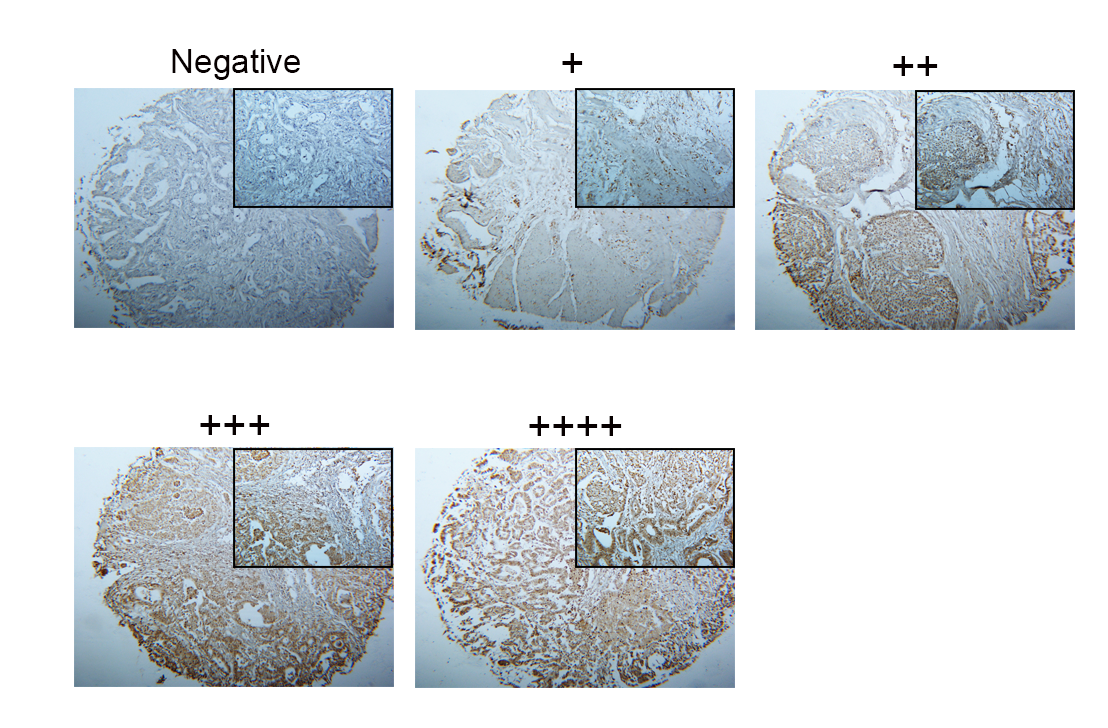


Supplementary Figure 1. **The grading standard of IL-17RB expression.**


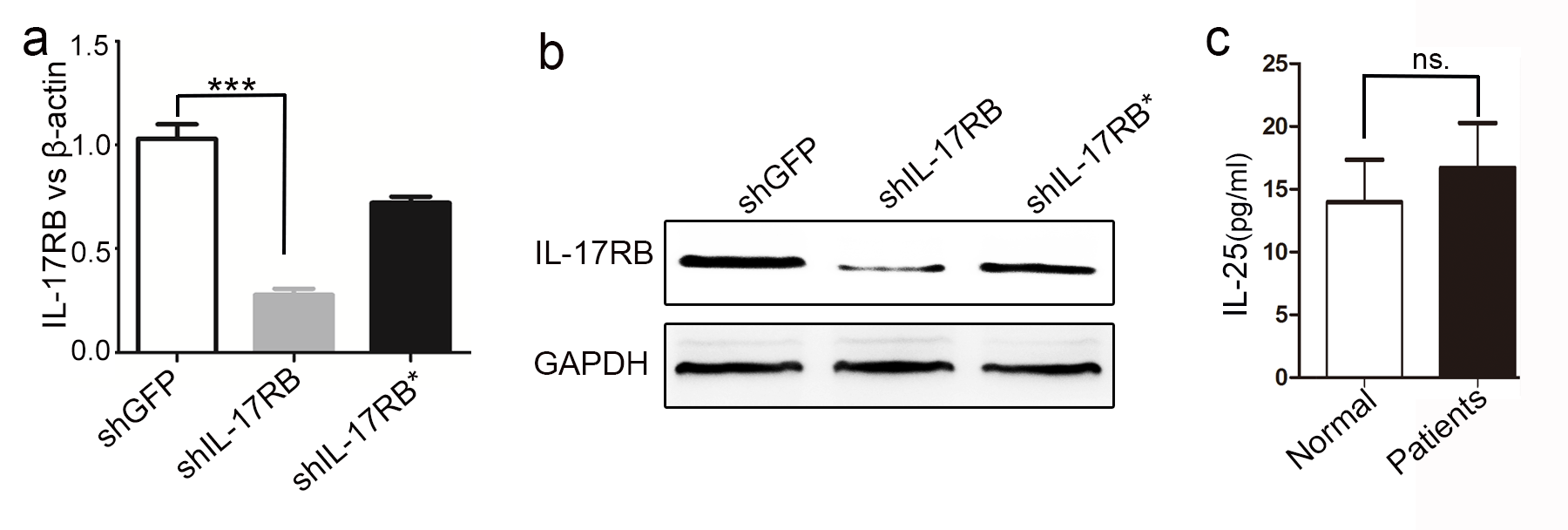


Supplementary Figure 2. **The expression of IL-17RB in MGC-803 transfected with shRNA and ELISA for serum IL-25.**

MGC-803 were transfected with IL-17RB-shRNA, IL-17RB^*^-shRNA or GFP-shRNA by lentivirus. The expression of IL-17RB in MGC-803 was determined by Real-time RT-PCR (a) and Western blot assay (b). (c): ELISA analyses the levels of IL-25 in the serum of gastric cancer patients and normal volunteers (n=60 vs 40; ns: No significant).


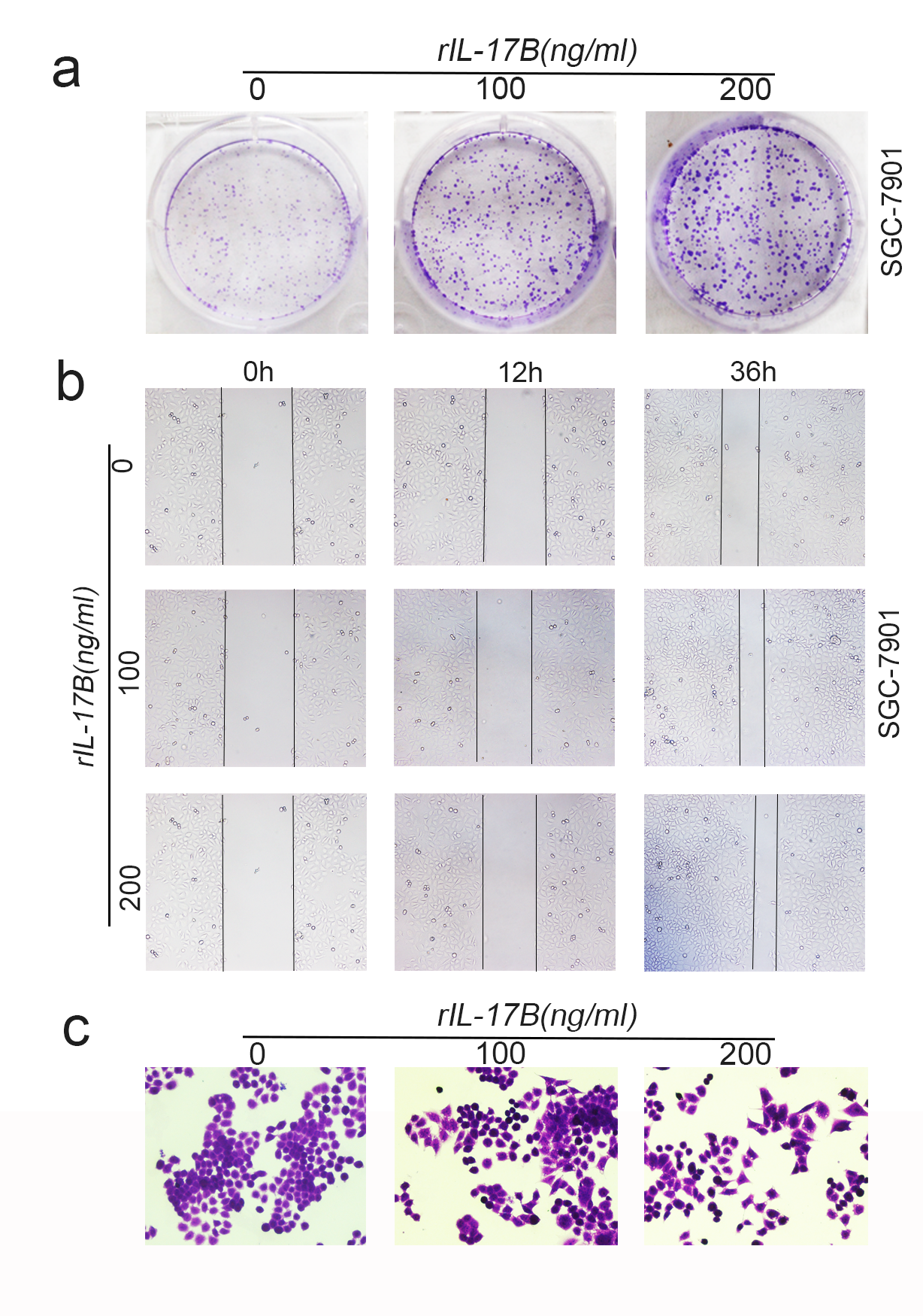


Supplementary Figure 3. **Exogenous IL-17B promotes the proliferation and migration of SGC-7901 cells and morphological changes of MGC-803.**

(a) Representative images of cell colonies in SGC-7901 cells treated with 100 ng/ml or 200 ng/ml IL-17B for 48h. (b) Representative images of the migration of SGC-7901 cells for 12 and 36 h treated with 100 ng/ml or 200 ng/ml IL-17B for 48h was detected by using cell scratch assay. (c) Representative morphological images of MGC-803 treated with 100 ng/ml or 200 ng/ml rIL-17B for 48h.


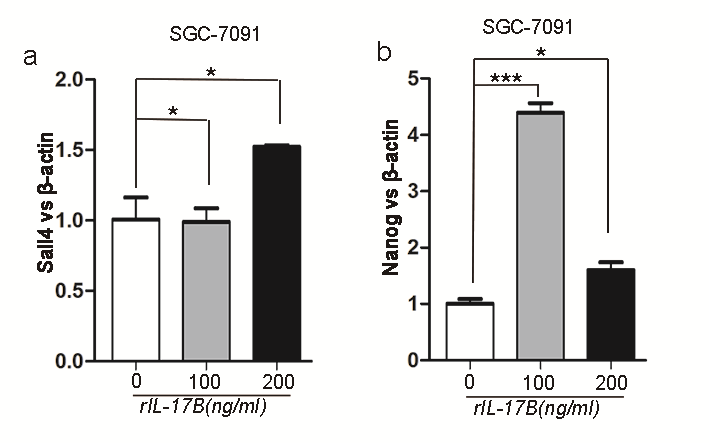


Supplementary Figure 4. **Exogenous IL-17B promote the expression of Sall4 and Nanog in SGC-7901 cells.**

Real-time RT-PCR analyses of Sall4 (a) and Nanog (b) in SGC-7901 treated with exogenous rIL-17B for 48h.


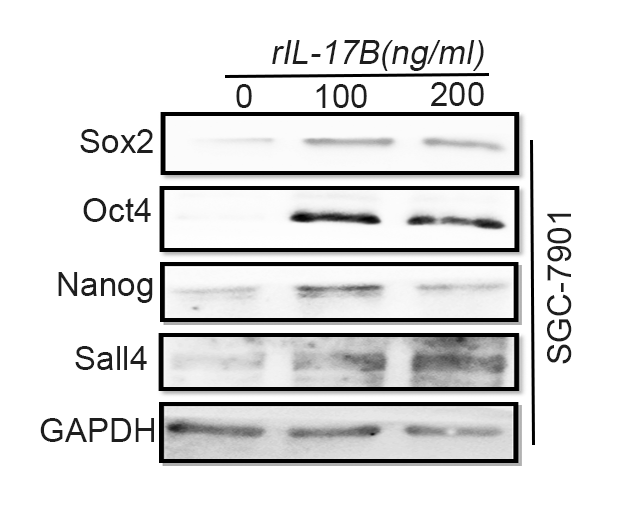


Supplementary Figure 5. **Exogenous IL-17B promote the expression of Sox2, Oct4, Nanog and Sall4 in SGC-7901 cells.**

The expression of Sox2, Oct4, Nanog and Sall4 was determined by Western blot assay in SGC-7901 cells treated with corresponding concentration of IL-17B for 48h.


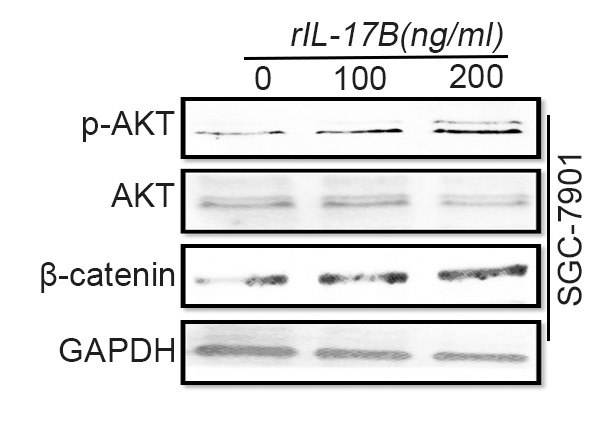


Supplementary Figure 6. **Exogenous IL-17B promote the phosphorylation of AKT and the expression of β-catenin in SGC-7901 cells.**

Western blot assay for p-AKT and total AKT, β-catenin in SGC-7901 cells treated with corresponding concentration of IL-17B for 48h.

Supplementary Table S1. Primer Sequences

| Target | Sequence (5’-3’) | Accession |
| --- | --- | --- |
| Human IL-17RB | Fwd: CTTGGTGGCCTTCAACAAGC | NM_018725' |
|  | Rev: AGAGCCGACCGTTCAATGTG |  |
| Human IL-17B | Fwd: GGTCCTCCTGCATGGTGAAG | NM_014443 |
|  | Rev: ACTGGCCTCACAACCTGCTG |  |
| Human IL-25 | Fwd: GGTAGAGCAGCTCCGAGTTG | NM_172314 |
|  | Rev: ACCAGGTGGTTGCATTCTTG |  |
| Human Nanog | Fwd: GCAATGGTGTGACGCAGAAG | NM_024865 |
|  | Rev: GCATGCAGGACTGCAGAGAT |  |
| Human Oct4 | Fwd: CAGAGTGGTGACGGAGACAG | NM_002701 |
|  | Rev: AGAAGGATGTGGTCCGAGTG |  |
| Human LGR5 | Fwd: ATGTTCACTGCTGCGATGAC | NM_003667 |
|  | Rev: AGGCTCAAGATGAACGTGAC |  |
| Human Sox2 | Fwd: ACACCAATCCCATCCACACT | NM_003106.3 |
|  | Rev: GCAAACTTCCTGCAAAGCTC |  |
| Human Sall4 | Fwd: TCGATGGCCAACTTCCTTC | XM_011528919.1 |
|  | Rev: GAGCGGACTCACACTGGAGA |  |
| Humanβ-actin | Fwd: TGGCACCCAGCACAATGAA | XM_005249820.1 |
|  | Rev: CTAAGTCATAGTCCGCCTAGAAGCA |  |

Supplementary Table S2. ShRNA and siRNA Oligonucleotides

| Target Gene | Sequences (5′-3 ′) |
| --- | --- |
| Human-shIL-17RB | Forward CCGGGATGCTACAACATGATCTAATCTCGAGATTAGATCATGTTGTAGCATCTTTTTG |
|  | Reverse  AATTCAAAAAGATGCTACAACATGATCTAATCTCGAGATTAGATCATGTTGTAGCATC |
| Human-shIL-17RB^*^ | Forward CCGGCCAGAACTGTTTAGCTAATATCTCGAGATATTAGCTAAACAGTTCTGGTTTTTG |
|  | Reverse AATTCAAAAACCAGAACTGTTTAGCTAATATCTCGAGATATTAGCTAAACAGTTCTGG |
| Human-shGFP | Forward CCGGGCAAGCTGACCCTGAAGTTCATCTCGAGATGAACTTCAGGGTCACGTTGCTTTTTG |
|  | Reverse AATTCAAAAAGCAAGCTGACCCTGAAGTTCATCTCGAGATGAACTTCAGGGTCACGTTGC |
| Human-siAKT | Forward GCGUGACCAUGAACGAGUUTT |
|  | Reverse AACUCGUUCAUGGUCACGCTT |
| GAPDH Positive control | Forward UGACCUCAACUACAUGGUUTT |
|  | Reverse AACCAUGUAGUUGAGGUCATT |
| Negative control | Forward UUCUCCGAACGUGUCACGUTT |
|  | Reverse ACGUGACACGUUCGGAGAATT |
